# Recent advancements in artificial intelligence applications for the mitigation of antimicrobial resistance: challenges and opportunities

**DOI:** 10.1093/jacamr/dlag144

**Published:** 2026-08-10

**Authors:** Swetha Valavarasu, Sandhya Amol Marathe, Sanjay Kumar Kochar, Tanmaya Mahapatra

**Affiliations:** Department of Computer Science and Information Systems, Birla Institute of Technology and Science, Pilani, Pilani Campus, Vidya Vihar, Pilani, Rajasthan 333031, India; Department of Biological Science, Birla Institute of Technology and Science, Pilani, Pilani Campus, Vidya Vihar, Pilani, Rajasthan 333031, India; Department of Medicine, Sardar Patel Medical College, Bikaner, Rajasthan 334001, India; Department of Computer Science and Information Systems, Birla Institute of Technology and Science, Pilani, Pilani Campus, Vidya Vihar, Pilani, Rajasthan 333031, India

## Abstract

Antimicrobial resistance (AMR) poses a significant global public health threat, and efforts to mitigate it have been aided by artificial intelligence (AI) methods. Key areas of applications include rapid diagnostics, drug discovery and repurposing, surveillance and predictive modelling, and antibiotic stewardship. This review aims to summarize key literature on AI applications for mitigating AMR including original research articles from PubMed and Scopus published from October 2024 to 2025 to summarize and find out the way ahead for successful application of AI. The key search terms included were AMR, AI and applications such as diagnostics, drug discovery and repurposing, surveillance, predictive modelling and stewardship. The data used for these applications, the techniques applied and the predictive targets have undergone significant expansion, along with the focus on model deployment, external validation and model interpretability. Although more complex models, such as neural networks and transformers, have been experimented with, classical machine learning (ML) models still dominate the AMR prediction space, while large language models are also being tested for prediction, as well as antibiotic stewardship. For drug discovery, data mining for antimicrobial peptides from different sources is a major application. Predictive modelling using next-generation sequencing data has been the most studied. The application of AI/ML to large and complex data from multiple sources could provide a promising arena for developing clinically translational tools. With more data availability, regulatory measures, real-world validation and transparency, there is scope for responsibly integrating innovative technology into clinical practice.

## Introduction

Antimicrobial resistance (AMR) has rapidly escalated into a global public health crisis that threatens the efficacy of antibiotics, the cornerstone of modern medicine. Once considered a distant, looming threat, AMR has been affecting the treatment of common infections and routine medical procedures, placing immense strain on the healthcare systems throughout the world. The increasing burden of morbidity and mortality caused by AMR is alarming. In the study by Antimicrobial Resistance Collaborators, 2022,^1^ it was estimated that bacterial AMR was directly attributable to around 1.27 million deaths and linked to nearly 4.95 million deaths in 2019 alone. These numbers are projected to rise dramatically if urgent and effective action is not taken. The World Health Organization (WHO) has warned that by 2050, AMR could result in 10 million deaths annually and cost the global economy up to USD 100 trillion in healthcare.^2^

With the advent of artificial intelligence (AI), which has emerged as a powerful resource in the multifaceted efforts to curb the emergence and spread of AMR, there are new avenues for utilizing available data to generate new insights. The momentum of AI in healthcare has been extended into the research and management of various aspects of AMR. AI can contribute across the entire AMR continuum, from diagnosis and treatment to surveillance and drug discovery.^3^ In diagnostics, AI algorithms can interpret complex biological data, enabling faster identification of pathogens and resistance determinants like mutations and antibiotic resistance genes (ARGs). Antibiotic stewardship could be optimized using AI for making prescribing decisions by integrating various data from patients, local resistance patterns and pharmacological knowledge. For monitoring and surveillance, AI finds its use in synthesizing different data streams to identify emerging resistance hotspots and predict the trends of resistance, to help maintain the efficacy of existing treatments. In drug development, AI helps in accelerating the discovery of new antimicrobials, combination therapies, antimicrobial peptides (AMPs) and adjuvants, and in repurposing existing compounds with potential antimicrobial properties.^4,5^

This review provides a comprehensive overview of the recent advancements in the various approaches to applications of AI in mitigating AMR over the past year. It begins with Section 2, with an exploration of AI-driven diagnostics, where technologies such as deep learning (DL) have enhanced the speed and accuracy of identifying resistant pathogens with inputs from microscopic imaging and molecular biology laboratory techniques. Then, Section 3 examines AI's role in drug discovery and repurposing, two critical areas given the urgent need for new antibiotics and the need to maintain the effectiveness of existing treatments. In Section 4, AI applications in AMR surveillance and predictive modelling have been discussed, followed by the integration of AI in antibiotic stewardship programmes in Section 5. This review provides a snapshot of the most recent advances made in clinical AI/machine learning (ML) applications focused on curbing and managing AMR. Summarized in Section 6, the trends of the data used, feature selection, organisms studied, validation techniques used, AI/ML model interpretability and model deployment have provided a broad overview of the current challenges and limitations. We propose potential future directions to develop robust AI tools that can be integrated into clinical practice.

## AI-driven rapid diagnostics

Conventional antimicrobial susceptibility testing (AST) methods, including broth microdilution or disk diffusion, typically require 24–72 h for definitive results.^6^ In recent years, the convergence of AI/ML methods with rapid diagnostic technologies has demonstrated transformative potential in accelerating the detection of resistant pathogens.^7^ This review synthesizes findings from the latest studies examining AI/ML applications in AMR diagnostics, emphasizing neural network architectures, interpretability frameworks and emerging models deployed in real-world clinical environments.

AI-driven systems offer an alternative to the time-consuming conventional methods by leveraging electronic health records (EHRs), microscopic imaging, spectroscopy and genomic data to provide rapid and real-time diagnostics.^8^ These help accelerate processes through image analysis, genomics and point-of-care integration. AI-powered diagnostics also aid in reducing empirical treatment, improving therapeutic outcomes and monitoring resistance development.

ML-assisted analysis of matrix-assisted laser desorption/ionization time-of-flight mass spectrometry (MALDI-TOF MS) data has emerged as a promising strategy for rapid detection of AMR in clinically important bacterial pathogens. Several recent studies have explored its application for predicting resistance in methicillin-resistant *Staphylococcus aureus* (MRSA), *Klebsiella pneumoniae*, *Mycobacterium abscessus*, *Escherichia coli*, *Burkholderia pseudomallei* and *Staphylococcus epidermidis*.^9–18^ By identifying spectral signatures associated with resistant phenotypes, these approaches have the potential to generate susceptibility-related information significantly earlier than conventional culture-based antibiotic susceptibility testing, thereby enabling optimization of antibiotic therapy and reducing reliance on empirical broad-spectrum antibiotic use. The feasibility of this work was studied by Weis *et al*., who demonstrated that ML models trained on MALDI-TOF MS spectra could directly predict antimicrobial susceptibility from clinical bacterial isolates, with accuracies close to those of phenotypic tests, highlighting the translational potential of integrating AI into routine microbiology workflows.^19^ Building on this, Yong *et al*. developed a randomized multicentre ML framework for rapid MRSA screening, representing one of the strongest validations of MALDI-TOF-based AI diagnostics across diverse clinical settings.^9^ Similarly, Xu *et al.* demonstrated rapid detection of carbapenem-resistant *E. coli* and *K. pneumoniae* directly from positive blood cultures, using tree-based ML models, suggesting potential utility in reducing delays in initiating effective therapy for severe infections.^15^

Recent work has also expanded beyond resistance prediction towards decision-support applications. Lin *et al*. and Jian *et al*. developed AI-enhanced clinical decision-support systems (CDSS) that integrate MALDI-TOF MS data to accelerate antibiotic selection and predict resistance in multidrug-resistant pathogens, illustrating how existing diagnostic platforms can be coupled with AI to support antimicrobial stewardship interventions.^10,13^ Likewise, De Waele *et al*. designed a dual-branch neural network-based antimicrobial drug recommender system that linked MALDI-TOF-derived pathogen information directly with antibiotic recommendations, moving beyond organism identification towards personalized treatment support.^12^ Gómez de la Torre *et al*. further showed that AI-based treatment recommendations derived from molecular and phenotypic data improved the speed and accuracy of bacteraemia management compared with conventional approaches, suggesting a growing role for AI-assisted therapeutic decision-making in clinical practice.^14^

Other studies have focused on extending MALDI-TOF MS applications to specialized pathogens and broader resistance prediction tasks. Thai *et al*. demonstrated discrimination between drug-susceptible and resistant strains of *M. abscessus*, while Nithimongkolchai *et al*. applied similar approaches for rapid resistance detection in *B. pseudomallei*, an important pathogen associated with melioidosis.^11,16^ Astudillo *et al*. developed multi-label classification models capable of simultaneously predicting resistance to multiple antibiotics from raw clinical MALDI-TOF spectra, while *Ren et al*. showed that MALDI-TOF MS platforms could be repurposed for resistance screening in *S. epidermidis*.^17,18^ Collectively, these studies demonstrate substantial progress towards integrating AI-assisted MALDI-TOF diagnostics into microbiology laboratories; however, despite encouraging performance, challenges related to prospective multicentre validation, workflow integration, regulatory approval and ensuring safe clinical deployment remain important barriers before widespread routine implementation.

Fourier transform infrared (FTIR) spectroscopy combined with ML has also emerged as a promising tool for rapid bacterial characterization and resistance detection. In a multicentre study, Candela *et al*. developed an FTIR-based model using principal component analysis and ML algorithms for typing carbapenemase-producing *Klebsiella pneumoniae*, with artificial neural networks achieving the highest accuracy (80.5%).^20^ Although this approach does not directly predict AMR, rapid strain typing may support outbreak detection, infection control and surveillance of high-risk resistant pathogens in healthcare settings. Bonner *et al*. further demonstrated that FTIR-based biochemical profiling combined with ML could provide early indications of phenotypic resistance development within 24 h of antibiotic exposure, achieving accuracies of up to 96%.^21^ Such approaches may help identify emerging resistance earlier than conventional susceptibility testing and support more timely therapeutic decision-making. However, despite encouraging performance, FTIR-based AI systems remain largely investigational and require broader clinical validation before routine implementation.

ML models using liquid chromatography-mass spectrometry (LC-MS) data have also been explored for rapid bacterial characterization and resistance detection. Olajide *et al*. demonstrated that ML-assisted LC-MS analysis could distinguish 11 *E. coli* strains in urine samples based on biochemical signatures, highlighting its potential utility for rapid bacterial identification and strain differentiation, particularly in epidemiological investigations, although its direct implications for antimicrobial stewardship remain limited.^22^ Dixon *et al*. further applied supervised ML to metabolomic data to distinguish carbapenemase-producing Enterobacterales (CPE) from non-CPE isolates within 7 h, achieving an AUROC (area under the receiver operating characteristic curve) of 0.85.^23^ Earlier identification of carbapenem resistance may help clinicians initiate effective therapy sooner, although these metabolomics-based approaches require further validation before routine diagnostic adoption.

Raman spectroscopy combined with ML has also been investigated for rapid bacterial identification and characterization. Lin *et al*. developed AIRDIS (artificial intelligent raman detection and identification system), which differentiated clinically important pathogens including *S. aureus*, *Enterococcus faecium*, *K. pneumoniae*, *Pseudomonas aeruginosa* and *Acinetobacter baumannii* with accuracies of 94.5%–97.6%, demonstrating the potential of AI-assisted spectroscopy for faster organism identification.^24^ Fernández-Manteca *et al*. used DL to identify hypermucoviscous *K. pneumoniae* capsular serotypes with high accuracy, which may be valuable for epidemiological surveillance and outbreak investigations, although its direct contribution to antimicrobial stewardship is less clear.^25^ Liu *et al*. further developed a DL-based Raman platform capable of rapid bacterial classification using intracellular biomolecular signatures, illustrating how AI-integrated spectroscopy may reduce diagnostic turnaround times.^26^ However, most Raman-based systems remain investigational and require further clinical validation before integration into routine microbiology laboratories.

DL models have also been applied to automate the interpretation of AST images. Nguyen *et al*. developed image-based DL systems, including disc diffusion reader and object detection models, to automate Kirby-Bauer disk diffusion interpretation, potentially reducing manual reading variability and improving access to susceptibility testing in resource-limited settings.^27,28^ Farrar *et al*. further demonstrated that convolutional neural network (CNN) analysis of fluorescent ribosomal microscopy images could distinguish susceptible and resistant *E. coli* strains within 30 min of antibiotic exposure, substantially reducing the time required compared with conventional culture-based testing.^29^ Such approaches may accelerate susceptibility reporting and support earlier targeted therapy, although workflow integration and validation in routine diagnostic laboratories remain necessary.

AI has also been integrated into molecular diagnostic platforms for rapid detection of resistance genes. Castellanos *et al*. developed an ML-assisted platform combining sample lysis, gene amplification and smartphone-based automated interpretation for rapid detection of extended-spectrum beta-lactamases (ESBL) and carbapenemase genes in Gram-negative bacteria called URECA-LAMP (URine, ESBL, and CArbapenemase Loop-mediated isothermal AMPlification), with accuracy exceeding 95%.^30^ Such portable systems may improve access to rapid resistance diagnostics, particularly in low-resource settings where conventional molecular testing infrastructure is limited. Another study using ESBL genes attempted to validate generative pre-trained transformers (GPTs)—GPT-4 and GPT-agent for pre-classification of disk diffusion to indicate potential beta-lactamases in 225 Gram-negative isolates. Compared to GPT-agents, humans (microbiologists) showed higher specificities, and GPT-agents’ unspecific identification of ESBL and AmpC beta-lactamase genes could potentially lead to additional testing, delays in diagnosis and higher costs. Also, GPT-4 is not approved by regulatory bodies, and large language model (LLM) validation is essential to assess its utility in clinical settings.^31^

## AI-driven drug discovery and repurposing

AI/ML methods have been utilized to efficiently screen through the chemical landscape of potential antibiotics across drug repurposing, *de novo* design and mechanism discovery.^4^ Repurposing existing drugs to bypass preclinical safety hurdles is an effective strategy due to cost-effectiveness, known pharmacokinetic profiles and accelerated translation. Recently, a DL-guided study identified Efavirenz, an antiretroviral agent, as a potential antibacterial lead targeting *S. aureus* and *Enterococcus* spp.^32^  *In silico* binding models predicted high affinity for penicillin-binding proteins and the WalK membrane kinase, later validated through synergistic *in vitro* assays.

Using a DL model trained to predict antimicrobial activity from molecular structure, Stokes *et al.* identified halicin, originally developed as an anti-diabetic agent, as a promising antibiotic candidate. Subsequent experimental validation confirmed broad-spectrum activity against multiple multidrug-resistant pathogens in mice.^33^ Although halicin remains one of the most widely recognized examples of AI-assisted antibiotic discovery, its development has thus far been limited to preclinical evaluation and has not progressed to clinical use. In a recent study, researchers tested the drug's antibacterial activity against multidrug-resistant bacteria and found notable inhibition of bacterial growth in *E. coli* and *S. aureus*, especially when combined with other drugs, while *P. aeruginosa* exhibited intrinsic resistance. Further clinical investigations are necessary to assess its safety and therapeutic application.^34^ Furthermore, pre-trained molecular embedding models such as MolE (molecular representation through redundancy reduced embedding), a self-supervised DL framework, have shown that unsupervised learning from millions of chemical structures can generalize antimicrobial predictions across drug classes, enabling *de novo* identification of antibacterial potential in human-targeted drugs.^35^ AI has helped uncover hidden antibacterial scaffolds by learning physicochemical and topological features linked to bacterial inhibition. These findings highlight the growing role of AI in prioritizing promising antibacterial candidates more efficiently than conventional screening approaches. However, extensive pharmacological characterization and safety validation remain necessary before therapeutic translation.

AMPs are a potential class of therapeutics for which AI has been used to navigate through the vast sequence space effectively for mining and generation.^36^ These could serve as templates to overcome resistance through intracellular targeting or membrane disruption, though issues such as cytotoxicity and instability have limited their therapeutic use. New DL architectures have transformed peptide discovery. Efficient *de novo* Broad-spectrum Antimicrobial Peptide discovery framework (EBAMP), a generative-discriminative transformer framework, produced 256 peptides with 37.5% showing bactericidal activity against resistant bacteria and fungi. Lead peptides further showed potent activity [minimum inhibitory concentration (MIC) as low as 2 mg/L], broad-spectrum activity, low toxicity with minimal drug-resistance risk and membrane-disruptive antibacterial mechanisms.^37^ UniProtKB/Swiss-Prot database mining identified over 8000 novel eukaryotic AMPs via the AMPlify model.^38^ Thirteen out of the 38 synthesized peptides with similarities to known chicken AMPs showed antimicrobial activity against *E. coli* and *S. aureus*. The AMP-Designer foundation model leveraged a LLM to generate AMPs *de novo* within 11 days, achieving a 94% experimental success rate and nanomolar potency *in vivo*.^39^ Biodiversity mining using large-scale genomic and proteomic datasets from marine cnidarians, ruminant microbiomes, venoms and Lactobacillaceae genomes, each powered by DL filters, has yielded tens of thousands of new AMP candidates (summarized in Table 1).^40–43^ Aggregation-prone AMPs from octopus proteins exemplify how AI can uncover noncanonical intracellular mechanisms, such as nucleic acid aggregation and translation inhibition. The lead peptide Oct-P2 exhibited potent antibacterial activity with minimal cytotoxicity in mammalian macrophage cells, indicating favourable selectivity for therapeutic development.^44^ Collectively, these studies demonstrate how AI-driven multi-omics mining extends beyond chemical space into biological diversity, redefining the boundaries of antimicrobial discovery. These advances converge on an emerging model of closed-loop discovery: design or predict candidates using AI, validate them using experimental assays and retrain the model based on the feedback. Such iterative systems may accelerate optimization of peptide candidates during early-stage discovery. Nevertheless, challenges related to peptide toxicity, instability, manufacturing scalability and limited *in vivo* persistence continue to restrict the clinical translation of many AI-designed AMPs.

**Table 1.** Selected examples of AMP mining using AI/ML methods

Sl. no.	AMPs designed	Source	Method used	Target pathogens	Properties	References
1	Generated 256 AMPs, out of which 96 show bactericidal activity	UniProt database (pre-training)	Transformer-based generative models	*A. baumannii*, *E. coli*, *K. pneumoniae*, *P. aeruginosa*, *S. aureus*, *Candida auris* and *Candida albicans*	Low MIC (2 mg/L), low toxicity/drug-resistance risk, broad spectrum, disrupts cell membrane structure	37
2	Identified 8008 novel putative AMPs, out of which 13 out of 38 prioritized ones were synthesized	UniProtKB/Swiss-Prot database	Attention-based DL	*E. coli* and *S. aureus*	Potential applications in chicken farming; AMPlify workflow	38
3	∼3130 unique AMP candidates derived from 111 species of phylum Cnidaria	Omics data (proteomes + transcriptomes) from 111 Cnidaria species	*In silico* proteolysis to generate virtual peptidomes; then consensus of multiple ML prediction tools for AMP activity and toxicity; followed by complex-network/chemical-space analysis to filter and cluster the candidates	*Bacillus subtilis*, *E. coli*, *K. pneumoniae*, *P. aeruginosa* and *S. aureus*	Non-haemolytic, non-toxic	40
4	27 192 potential secretory AMP candidates were initially predicted; 39 selected for synthesis; one (peptide 4) shown active against MRSA *in vivo*	Ruminant gastrointestinal microbiome datasets (120 metagenomes + 10 373 metagenome-assembled genomes)	DL (Natural Language Processing algorithms) for AMP classification	Primarily MRSA, also tested on *S. aureus*, *S. agalactiae*, *E. coli* and *S. enterica*	• High hit-rate: all 39 synthesized peptides showed antimicrobial activity versus *S. aureus*; ∼79.5% were effective against multiple pathogens • One lead (peptide 4) showed *in vivo* efficacy in a mouse wound infection model with MRSA • Low cytotoxicity and low haemolysis • Mechanism: membrane disruption (as shown by molecular dynamics simulations)	41
5	Venom-encrypted peptides (VEPs) mined from global venom proteomes: 16 123 venom proteins were explored, from which ∼40 626 260 peptide fragments were generated. Three hundred and eighty-six novel candidates were selected, of which 58 were synthesized and 53 validated	Venom protein databases: e.g. ConoServer (cone snail peptides), Arachnoserver (spider venoms), ISOB (indigenous snake proteins), VenomZone (multi-taxon venom proteins)	DL models to predict MIC values	Eleven clinically relevant pathogens including *E. coli*, *K. pneumoniae*, *S. aureus* and antibiotic-resistant strains (Gram-negative and Gram-positive bacteria)	Low cytotoxicity; lead VEPs structurally and functionally different from known AMPs	42
6	Nine thousand six hundred and one AMPs were identified, out of which 1516 gene cluster families were novel	Lactobacillaceae genomes from NCBI	Pre-trained DL model (APEX v2) to predict MIC values	Eleven clinically important Gram-positive and Gram-negative bacteria	Broad spectrum (especially delbruin_1); some of the experimentally tested AMPs showed high specificity to target pathogens and narrow-spectrum potential as anti-infectives	43
7	dHP3-31, dHP3-50, dHP3-50.137, dHP3-50.190, dHP3-84 and dHP3-84.39	Frog skin	ML models for bioactivity prediction	*E. coli*, *S. aureus*, *K. pneumoniae*, *A. baumannii* and *S. mutans*	Reduced cytotoxicity on eukaryotic cells, higher selectivity and therapeutic indices	44

ML has revitalized quantitative structure–activity relationship (QSAR) modelling by learning non-linear relationships between molecular descriptors and antimicrobial potency. AI-augmented rational design improved frog-skin-derived AMPs by optimizing hydrophobic and cationic balance for enhanced Gram-negative activity. Importantly, the optimized peptides demonstrated reduced cytotoxicity towards eukaryotic cells, greater target selectivity and improved therapeutic indices, highlighting their enhanced safety and translational potential.^45^ Similarly, datasets combining QSAR and ML models (random forest, logistic regression) have enabled the screening of β-lactamase inhibitors from large compound libraries for structure–activity prediction.^46^ In addition, perturbation theory ML for design of inhibitors against *S. aureus* has achieved more than 80% prediction accuracy for multi-strain inhibitors and graph convolutional models have revealed key molecular fragments driving antibacterial activity for predicting antimicrobial efficacy by integrating three types of molecular fingerprints along with molecular graph representations.^47,48^ AI bridges the gap between data-driven models and chemical intuition by highlighting key functional groups, charge distribution and fragment contributions that influence antimicrobial behaviour.

The lack of mechanistic transparency of candidate molecules remains a bottleneck for regulatory approval. The HoloMoA framework integrates time-lapse digital holographic microscopy with DL to classify antibiotic MoAs (mechanism of action) with 95% accuracy and identify potential novel mechanisms. By coupling phenotypic imaging and convolutional recurrent neural networks, such methods allow rapid MoA inference, reducing dependency on labour-intensive molecular assays.^49^ Such approaches may improve understanding of how candidate compounds affect bacterial cells, which is important for prioritizing promising molecules during early-stage drug discovery. However, translating these findings into clinically approved therapies remains a long and highly regulated process.

Researchers developed an ML-driven framework integrating data from a large antimicrobial knowledge graph. This graph serves as a structured repository that aggregates and visualizes public *in vitro* antibacterial assay data. Using this dataset, seven ML models were trained across six molecular fingerprint representations to predict antimicrobial activity. The random forest model using MHFP6 fingerprints achieved the best performance (accuracy 75.9%, Cohen's kappa 0.68). The model's predictive capacity was validated by screening the EU-OpenScreen and Enamine compound libraries, correctly identifying over 30% of active compounds and predicting pathogen class specificity. This study demonstrates how combining knowledge graphs with ML-driven prediction can streamline antimicrobial screening, prioritize active candidates and reduce discovery costs, offering a scalable approach to accelerate AMR drug discovery and compound repurposing.^50^ Despite these advances, the majority of AI-driven antimicrobial discovery studies remain focused on computational prediction, laboratory validation or preclinical testing. Among currently reported candidates, only a limited number, such as halicin and recently developed AI-designed AMPs, have progressed to advanced preclinical evaluation, while translation into human clinical trials remains largely absent.

## AI in AMR surveillance and predictive modelling

Prediction of AMR from various data sources, such as clinical indicators from EHRs, phenotypic susceptibility testing and genomics, has been well studied.^8^ Some of the notable additions include ML models such as LightGBM (light gradient-boosting machine) and XGBoost (extreme gradient boosting) using large datasets such as EHR data from STARR (Stanford Translational and Administrative Research Repository), and Pfizer ATLAS (Antimicrobial Testing Leadership and Surveillance) Antibiotics to predict susceptibility to antibiotics for enhancing stewardship in inpatient care and surveillance, respectively.^51,52^ Other studies using retrospective clinical data predict AMR in Enterobacterales bloodstream infections (BSIs)^53,54^ and intensive care units (ICUs)^55,56^ have been done, with emphasis on interpretability and deployment. Although these approaches have shown promising predictive performance, most models remain retrospective or research-stage tools, with relatively limited integration into routine clinical decision-making workflows.

One study focused on interpretability for transparent clinical decisions that has been conducted for the prediction of MDROs (multidrug-resistant organisms) in ICUs from multiple clinical indicators using neural networks and classical ML models.^56^ Another study utilized routine automated AST results to predict phenotypic ESBL confirmation using MIC values in *E. coli*, *K. pneumoniae* and *Proteus mirabilis*, employing random forest, logistic regression and XGBoost. Interpretability was assessed using SHAP (SHapley Additive exPlanations) analysis, which identified the most contributing features as the antibiotics against which the bacteria were tested. External validation showed AUROCs of 0.89–0.93 for each of the bacterial models.^57^ Researchers performed a retrospective study to predict risk factors associated with hospital-acquired drug-resistant pathogens in patients with viral respiratory tract infections using seven ML models, with neural networks performing the best and SHAP analysis revealing length of stay, cholinesterase and age as the top features for prediction. Cholinesterase, which was tested for liver function, was a novel predictive indicator, with its decrease associated with increased risk of drug-resistant pathogen infections or colonization, possibly linked to the cholinergic anti-inflammatory pathway.^58^ Another study focused on hospital-acquired infections that has been done for the prediction of MDRO infections in patients with hospital-acquired pneumonia in the NICU (neuro-ICU). Logistic regression was the best-performing model (AUROC 0.805), with model interpretation identifying prolonged NICU stay, antibiotic exposure, diabetes and renal/metabolic biomarkers such as serum urea (described as carbamide in the original study) as important predictors. Such targeted interventions could help in infection risk prediction and in formulating antibiotic treatment.^59^ However, from a clinical perspective, reliance on predictive models without prospective validation may introduce risks such as inappropriate antibiotic escalation or delayed treatment if predictions are inaccurate, emphasizing the need for cautious implementation.

A study conducted in Germany attempted to analyse antibiotic resistance with emphasis on the effect of antibiotic consumption from ecological and clinical data using methods such as linear regression and neural networks. However, the study highlighted that inconsistent or incomplete surveillance data can substantially limit model reliability, underscoring the dependence of AI-based AMR prediction systems on high-quality longitudinal data collection.^60^

Next-generation sequencing (NGS) data have been utilized for AMR prediction due to the advantages it provides with respect to reduced time and better understanding of genetic factors responsible for resistance.^61^ Recent studies focus on a range of NGS data from various bacteria and different environments, providing evidence for the applicability of AI/ML techniques in combination with sequencing technologies to improve prediction capabilities. Increasing efforts have focused on developing interpretable AI tools that allow researchers and clinicians to better understand how genomic features influence resistance predictions, an important consideration for building trust in these systems.^62–67^ Earlier studies largely focused on ESKAPEE (*E. faecium, S. aureus, K. pneumoniae, A. baumannii, P. aeruginosa, Enterobacter species,* and *E. coli*) pathogens using genomic features such as single-nucleotide polymorphisms, k-mers and ARG (AMR gene) presence/absence patterns to predict resistance phenotypes.^7^ Recent work has expanded these approaches to other high-priority pathogens, including *A. baumannii*, *S. aureus*, Streptococcus and *E. coli*,^68–71^ while also extending predictive modelling to organisms such as *Enterobacter cloacae*, *Stenotrophomonas maltophilia* and *Listeria monocytogenes*.^72–74^ Among bacterial pathogens, *Mycobacterium tuberculosis* remains the most extensively studied, with models leveraging genomic variants, k-mer-based representations and resistance-associated mutations to predict susceptibility and improve rapid detection of drug-resistant tuberculosis.^67,75–82^ Apart from the novelty in the organisms studied, some studies have used novel methods. Some notable ones include the prediction of promoters in *Agrobacterium* and *Klebsiella* using novel feature engineering and an ensemble learning approach.^83^ One study has utilized whole-genome sequencing and metagenomic next-generation sequencing (mNGS) data for susceptibility prediction in *E. cloacae* with >95% accuracy.^72^ Models based on short-read mNGS for five of the ESKAPEE pathogens have been developed with ∼94% accuracy to predict AMR using mNGS-based AST data. The clinical utility of these models is yet to be validated, but they demonstrate the potential of ML models using diverse data inputs.^84^ In another study using multi-omics data to differentiate genes responsible for ceftazidime/avibactam and meropenem resistance in *P. aeruginosa*, the ML models were able to make the differentiation based on gene expression and protein abundances. This study also provided molecular insights into the molecular response of *P. aeruginosa* to the antibiotics, ceftazidime/avibactam and meropenem along with insights into bacterial molecular responses to antibiotic exposure, which may help guide future therapeutic strategies for managing resistant infections.^85^

In ML models predicting AMR from metagenomic sequences, several significant improvements have been made. Two of the studies utilizing faecal microbiome for prediction of antibiotic-resistant event outcomes in AML (acute myeloid leukaemia) patients and of checkpoint inhibitor efficacy in lung cancer patients are notable and represent the importance of integration of multiple data types to inform personalized therapy in vulnerable patients.^86,87^ Researchers have developed a rapid and interpretable rule-based genotypic model using minimal genomic determinants for *S. aureus*, with over 97% accuracy and emphasis on interpretability.^88^ A tool called DRAMMA has been developed to predict novel ARGs, without the need to rely on known genes from homology-based sequence similarities, enabling early detection of novel resistance genes.^89^ In another study using random forest models, researchers have been able to predict horizontal ARG transfer between diverse bacteria, utilizing a robust integrated dataset of metagenome sequences from various sources with an AUROC of 0.87.^90^ Although most of the models use random forests, decision trees, ensembles and neural networks, LLMs have also been increasingly utilized for AMR prediction models.^66,76,91^ Despite rapid advances in predictive modelling, the majority of AMR prediction systems remain largely confined to retrospective validation studies using curated datasets^51–56,58,59,86^ or bioinformatics pipeline and computational tool development.^63–66,74,89^ Comparatively, few studies have advanced towards prospective validation, clinical workflow integration or diagnostic implementation,^53,54,57,84,88^ with regulatory approval and real-world deployment still largely absent across the field. Challenges, including dataset heterogeneity, limited external validation, model interpretability and the risk of incorrect predictions influencing antibiotic prescribing decisions, remain important barriers to widespread clinical implementation.

## AI in antibiotic stewardship

Integration of AI/ML methods such as decision trees, boosting, and neural networks in antibiotic stewardship efforts has been described previously.^92,93^ CDSS represent one of the most actively explored applications of AI in antibiotic stewardship. These systems utilize ML algorithms to analyse large volumes of patient-specific data such as diagnosis, laboratory findings, microbiology results and pharmacokinetic/pharmacodynamic profiles to provide personalized treatment recommendations. When integrated into hospital EHRs, CDSS can support physicians by suggesting optimal empiric and targeted antibiotic therapies, flagging potential drug interactions and recommending appropriate de-escalation or discontinuation strategies.^94^ However, inaccurate recommendations or overreliance on automated decision-support systems may pose clinical safety risks, particularly in critically ill patients, emphasizing the need for physician oversight and prospective validation prior to routine implementation. Recent additions to these efforts include the utilization of EHR data for modelling AI/ML-based CDSSs and assisting prescription practices. In a study conducted in Anhui, China, random forest models were developed from routinely collected EHR data from around 7 million records of primary care visits over a period of 25 months from December 2019 to December 2021. The area under the curve values of the antibiotic prescribing models ranged from 0.92 to 0.97 for the seven disease diagnoses categories studied, with varying contributions from patient, clinician and spatiotemporal variables.^95^ Another use case analysis of the AI-CDSS system, KINBIOTICS, for antibiotic therapy in sepsis patients analysed the feasibility of the system and identified conditions that facilitate and inhibit its implementation in intensive care medicine by conducting expert interviews. The researchers employed a random forest model on patient, laboratory and clinical data, and interviewed physicians on their assessment of the pilot version, and found that variability in previous antibiotic administration practices affected the model's predictive ability.^96^ In the Netherlands, Dam *et al.* developed explainable ML models using data from 2486 ICU patients receiving antibiotic therapy to predict whether antibiotics discontinued during treatment would need to be restarted within 72 h after discontinuation. The models incorporated physiological monitoring data, laboratory parameters, microbiological culture results, selective decontamination strategies and medication records. Logistic regression was the best-performing model, achieving an AUROC of 0.67, while SHAP analysis identified penicillin use and the duration of the most recently prescribed antibiotic as the most influential predictors. The findings suggest that AI-assisted decision-support systems may help clinicians identify patients in whom antibiotic discontinuation is appropriate, thereby supporting antimicrobial stewardship efforts. However, prospective validation in real-world clinical settings is needed before routine implementation.^97^ Model interpretability remains particularly important for CDSS, as physicians must understand and trust the basis of model recommendations before incorporating them into patient care decisions.^98^

A more personalized treatment plan using ML models by identifying clinical factors for predicting vancomycin's steady-state trough concentration is important for maintaining therapeutic efficacy while minimizing toxicity. This is also a retrospective study involving 546 patients to whom intravenous vancomycin therapy was administered. Random forest models were developed using 57 clinical indicators and validated with internal and external datasets, as well as their performance compared with a Bayesian PopPK (Population Pharmacokinetics) model. The random forest models incorporated clinical indicators such as creatinine clearance, C-reactive protein, etc. and outperformed the Bayesian one with a predictive accuracy of 0.83. Such models may support individualized antibiotic dosing strategies and reduce the risk of underdosing or toxicity, although prospective clinical evaluation is required before routine integration into therapeutic drug monitoring workflows.^99^ Researchers have developed ML models to identify patient profiles with increased susceptibility to BSIs during the onset of febrile neutropenia (FN) in haematological malignancies, with the aim of tailoring antibiotic treatment approaches, especially with the rise of MDR infections. The study used an unsupervised ML algorithm, KAMILA (KAy-means for MIxed LArge data sets), to categorize the clinical phenotypes and determine BSI and MDR-Gram-negative bacteria prevalences at FN onset. The model's performance was validated with a cohort of data collected later, and the model clustered the data into three clusters: low, intermediate and high risk. This study found that ML-based risk stratification enhances data-driven decision-making for antibiotic therapy at FN onset.^100^

LLMs like ChatGPT have generated significant interest in their potential to assist in clinical decision-making, particularly in antibiotic prescribing. Several studies have assessed the ability of LLMs to guide physicians in selecting the appropriate antibiotics based on clinical data.^101,102^ Two recent studies that investigate the role of LLMs in antibiotic prescribing and resistance interpretation, comparing their performance to human experts, are discussed here. One study aimed to assess the performance of six different LLMs (ChatGPT, Gemini, Copilot, Mistral AI, Claude and Llama 3.1) in guiding antibiotic prescriptions for clinical vignettes that varied by infection type, patient demographics and comorbidities. The responses of LLMs were compared to national antibiotic prescribing guidelines across four countries: Ireland, the UK, the USA and Norway. The study also explored the challenges of LLMs, including hallucination, toxicity and data leakage. LLMs demonstrated high accuracy in diagnosing infections (92%–100%) and prescribing antibiotics (88%–100%). However, their adherence to national guidelines, particularly regarding drug choice, was less robust, with some LLMs referencing guidelines incorrectly (38%–96%), especially for Norway. Data leakage was also a concern, with personal information from vignettes being repeated in model outputs. In contrast, general practitioners referenced (100%) and prescribed (58%–92%) according to national guidelines; however, they showed inconsistency in the dose and duration of treatment (50%–75%). While LLMs are promising tools for guiding general antibiotic prescribing, they still fall short in applying national guidelines and determining the correct treatment. General practitioners remain better positioned to navigate these complexities.^103^

Another study evaluated the agreement between ChatGPT and infectious disease (ID) specialists regarding the selection of appropriate antibiotics and the interpretation of resistance mechanisms in 100 simulated clinical cases. The cases included detailed microbiological data and antibiograms, and both ChatGPT and ID specialists were tasked with recommending the best antibiotic and explaining the most likely mechanism of resistance. Agreement between ChatGPT and ID specialists was observed in 51/100 cases, with a Cohen's kappa coefficient of 0.48, indicating moderate agreement. However, agreement was lower for the interpretation of resistance mechanisms, with a kappa coefficient of 0.39 and agreement in only 42/100 cases. Levels of agreement varied based on the infectious syndrome and microorganism involved, suggesting that ChatGPT's performance is not consistent across different types of infections. ChatGPT displayed moderate agreement with ID specialists, but significant gaps remain in its ability to accurately recommend antibiotics and interpret resistance mechanisms.^104^ Although AI-driven stewardship systems have demonstrated encouraging performance across antibiotic prescribing, dosing optimization and treatment decision support, most studies remain retrospective or simulation-based. Variability in guideline adherence, inconsistent performance across clinical scenarios and the potential consequences of incorrect recommendations highlight the importance of maintaining clinician oversight and conducting prospective real-world validation before widespread implementation.

## Discussion

AI and ML have rapidly expanded across AMR-related diagnostics, drug discovery, surveillance and stewardship. Although this review highlights substantial methodological advances and growing translational interest, most AI applications in AMR remain at the proof-of-concept, retrospective validation or early pilot-testing stage, with relatively few systems having progressed to routine clinical implementation. Table 2 summarizes the major studies across the four domains reviewed: rapid diagnostics, drug discovery, surveillance and stewardship, along with key limitations that hinder deployment and generalizability. This consolidated overview provides a foundation for discussing the overarching challenges and future directions needed to strengthen AI-driven AMR solutions. The review included only English-language open-access publications. Consequently, relevant studies published in subscription-based journals or in other languages may have been missed, potentially introducing selection bias and limiting the comprehensiveness of the evidence base. Additionally, the rapidly evolving nature of AI research means that a substantial proportion of available evidence consists of early-stage methodological studies, which often emphasize algorithm development rather than long-term clinical or implementation outcomes.

**Table 2.** Summary of representative articles across categories with key contributions and limitations

Category	Representative articles	Key contributions	Key limitations
AI-driven rapid diagnostics	MALDI-TOF MS models (MRSA, *K. pneumoniae*, *E. coli*, etc.)^9–18^; FTIR-based early resistance detection^20,21^; Raman spectra-based DL models (AIRDIS, CNN, ResNet)^24–26^; microscopy-based CNN methods^27^; URECA-LAMP for ESBL/carbapenemase detection^30^; GPT-4-based ESBL classification pilot^31^	Rapid AST (minutes–hours), spectroscopic phenotyping, early prediction, point-of-care potential	Variability in sample preparation; small datasets; limited external validation; batch effects; expensive instrumentation; LLM risks (hallucination, non-regulatory compliance); limited clinical deployment
AI-driven drug discovery and repurposing	Efavirenz repurposing^32^; halicin follow-up studies^34^; MolE and self-supervised embedding models^35^; generative AMP platforms (EBAMP, AMPlify, AMP-Designer)^37–39^; biodiversity-driven AMP mining^40–43,45^; QSAR + DL for AMPs/β-lactamase inhibitors^44,46–48^; knowledge-graph-based antimicrobial screening^50^	Accelerated hit identification, structure–activity insights, generative peptide design, high *in vitro* hit rates, novel scaffolds	Lack of *in vivo* or clinical validation; toxicity/stability issues; limited mechanistic transparency; high false positives; high wet-lab cost; regulatory uncertainty for generative models
AI in AMR surveillance and predictive modelling	EHR-based models (STARR, ATLAS)^51,52^; BSI risk prediction^53–56^; ESBL phenotypic confirmation using ML^57^; hospital-acquired MDRO prediction^58,59^; ecological modelling of resistance versus consumption^60^; NGS-driven AMR prediction pipelines^62–84^; multi-omics resistance mechanism studies^85^; metagenomic risk stratification (AML, cancer)^86,87^; rule-based and interpretable genotype-to-phenotype models^88–90^	Demonstrated strong predictive performance within study datasets, scalable tools, interpretable SHAP-based insights, inclusion of novel pathogens	Data heterogeneity; geographic bias; limited multicenter datasets; complex, low-resource settings underrepresented; computational cost; lack of prospective testing
AI in antibiotic Stewardship (CDSS and prescribing)	EHR-based prescribing models in China^95^; KINBIOTICS feasibility study^96^; ICU antibiotic restart prediction^97^; vancomycin trough prediction^99^; FN risk clustering^100^; LLMs for prescribing (ChatGPT, Gemini, etc.)^101–104^	Demonstrated strong predictive performance under evaluated conditions, personalized dosing, risk stratification	Limited integration into workflows; inconsistent guideline adherence; interpretability gaps; LLM hallucination/toxicity; lack of regulatory approval; dependence on high-quality EHR data

Despite encouraging advances, several persistent challenges limit the real-world utility of AI-driven AMR solutions. Data fragmentation and heterogeneity remain significant obstacles: microbiology results, EHRs, sequencing data and environmental datasets differ substantially in structure, quality and availability, thereby reducing model generalizability across settings. Limited external and prospective validation is a consistent drawback, with many high-performing models failing when applied outside their development site. The lack of model interpretability, particularly in DL and LLM-based tools, undermines clinician trust and hinders regulatory acceptability. Additionally, algorithmic bias, including population underrepresentation (especially in low- and middle-income countries or LMICs), can lead to inequitable recommendations.^105^ From a regulatory standpoint, healthcare AI still lacks standardized evaluation pathways and monitoring frameworks after deployment, which complicates approval and implementation. Operationally, integrating AI tools into existing hospital workflows, EHR systems and stewardship programmes is resource-intensive. Overall, these limitations reinforce the need for robust, equitable and transparent AI pipelines. An important distinction must also be made between technical performance and clinical utility. Many studies report high predictive accuracy under controlled experimental conditions, yet relatively few demonstrate that these systems improve patient outcomes, reduce inappropriate antibiotic use, shorten time-to-effective therapy or meaningfully alter microbiology laboratory workflows. In several cases, AI models currently function primarily as decision-support or research tools rather than independently deployable clinical systems. Demonstrating real-world clinical benefit remains an important next step for the field.

From a clinician and healthcare worker perspective, the practical implications of AI-driven AMR tools extend beyond technical performance metrics.^106,107^ Incorrect AI recommendations may carry significant clinical consequences, including delayed initiation of effective therapy, inappropriate antibiotic escalation, unnecessary broad-spectrum antibiotic use or failure to detect resistant pathogens. These risks are particularly important in high-acuity settings such as ICUs and sepsis management, where timely and accurate antimicrobial decisions are critical. In addition, limited model transparency and poor integration into existing clinical workflows may reduce clinician confidence and hinder adoption. For this reason, maintaining clinician oversight remains essential to ensure that AI functions as a decision-support tool rather than a replacement for clinical judgment. This approach is consistent with emerging international frameworks for clinical AI governance, including WHO ethical guidance, human-in-the-loop decision models and regulatory standards emphasizing transparency, accountability and patient safety in healthcare AI deployment.^108,109^

To overcome these barriers, future research must prioritize the development of robust, generalizable and interpretable models. Large-scale, multicenter datasets, augmented through privacy-preserving methods such as federated learning, would improve representativeness and reduce geographic bias. Explainable AI frameworks, including SHAP, rule-based models and causal inference, should be embedded at the model design stage to enhance clinician trust and regulatory compliance. Strengthening prospective and real-time evaluation within routine clinical workflows is essential to bridge the gap between algorithmic success and clinical adoption. Additionally, integrating One Health datasets (human, animal and environmental) will enable more holistic modelling of resistance dynamics.^110^ There are significant opportunities in integrating transformer-based multimodal architectures, generative chemistry for novel therapeutics and AI-enhanced phenotypic MoA inference to accelerate both diagnostic and drug discovery pipelines. However, emerging generative AI systems, particularly LLMs, require careful evaluation because issues such as hallucination, lack of explainability and inconsistent adherence to clinical guidelines may compromise safe deployment in healthcare settings. Investments in computational and clinical capacity, especially in LMICs, are critical to ensure equitable benefit distribution and global readiness.^111^ However, in many resource-limited settings, the impact of AI-driven interventions may remain constrained in the absence of strong healthcare systems. Strengthening antimicrobial stewardship programmes, expanding AMR surveillance capacity, improving implementation of AMR policies and investing in the healthcare workforce and laboratory infrastructure may currently provide more immediate and sustainable benefits for AMR mitigation than widespread adoption of advanced AI technologies. This is particularly important because the burden of AMR disproportionately affects resource-limited settings, where clinicians empirically prescribe antibiotics due to limited laboratory testing, leading to antibiotic misuse or overuse, yet these regions remain underrepresented in the datasets used to train most contemporary AI systems.^1,112^ The simultaneous evolution of regulatory frameworks, particularly for generative AI applications, alongside the growth of AI technologies, is crucial to ensure the safe clinical use of these technologies.

While AI is not a one-stop solution for AMR, it offers an important opportunity to strengthen global efforts in diagnostics, therapeutic discovery, surveillance and stewardship. However, its long-term impact will depend not only on algorithmic innovation but also on rigorous clinical validation, regulatory oversight, equitable access and careful integration into existing healthcare systems. When developed and deployed ethically, transparently and collaboratively with clinicians and public health stakeholders, AI may become an important component of future global AMR mitigation strategies. The convergence of technological innovation, policy development and healthcare infrastructure will ultimately determine its contribution to preserving antibiotic effectiveness for future generations.

## Materials and methods

Research articles using relevant keywords were retrieved from PubMed and Scopus, with limitations on the English-language, open-access, humans and publications from the past year (October 2024–2025). Review articles and articles not using AI/ML were excluded from the analyses. The key search terms included were AMR, AI and applications such as diagnostics, drug discovery and repurposing, surveillance, predictive modelling and stewardship. The original research articles retrieved were screened and reviewed to gain insights into recent trends in input data, methodology and AI/ML model interpretation and deployment for efforts aimed at mitigating AMR.

## Conclusions

This review was written to capture the efforts made towards AMR mitigation over the past year, utilizing AI/ML strategies on various input data and prediction variables, particularly in the context of rapid diagnostics, drug discovery and repurposing, surveillance, predictive modelling and stewardship. Various methods, including ML, DL (neural networks), transformers and LLMs, have been utilized for a spectrum of applications, including mining AMPs, predicting resistance in bacteria from different sources, rapidly diagnosing resistant infections and aiding in antibiotic prescription. The shift to more studies incorporating model deployment, external validation and ensuring model interpretability is promising to the translational impact that large-scale data can have, especially in clinical settings. The use of new data inputs or their combinations, prediction models, prediction variables, feature selection, organisms studied, patient cohorts and model implementations displays the scope and potential of well-designed AI/ML models in AMR prediction in both clinical and environmental settings.

## Data Availability

No new data were created or analysed in this study. Data sharing is not applicable to this article.
